# Twenty years’ experience of type B aortic dissections: a population-based national registry study from Finland

**DOI:** 10.1093/icvts/ivad184

**Published:** 2023-11-08

**Authors:** Juhana Mikael Toimela, Jagdeep Sedha, Marja Hedman, Antti Valtola, Tuomas Selander, Annastiina Husso

**Affiliations:** Department of Cardiothoracic Surgery, Kuopio University Hospital, Kuopio, Finland; Department of Medicine, University of Eastern Finland, Kuopio, Finland; Department of Cardiothoracic Surgery, Kuopio University Hospital, Kuopio, Finland; Institute of Clinical Medicine, University of Eastern Finland, Kuopio, Finland; Department of Cardiothoracic Surgery, Kuopio University Hospital, Kuopio, Finland; Science Service Center, Kuopio University Hospital, Kuopio, Finland; Department of Cardiothoracic Surgery, Kuopio University Hospital, Kuopio, Finland

**Keywords:** Aortic dissection, Type B, Incidence, Thoracic endovascular aortic repair, Aortic surgery

## Abstract

**OBJECTIVES:**

The objective of this study was to investigate the incidence, treatment and survival of Stanford type B aortic dissection (BTAD) during 20 years in the Finnish population.

**METHODS:**

Data collection was made from the Nationwide Care Register for Health Care, Finnish National Institute for Health and Welfare. All patients over 15 years of age with BTAD from 2000 to 2019 were included in the study. A data search of the Registry of Death Cause (Statistic Finland) was carried out to identify the date and cause of death.

**RESULTS:**

There were 1742 cases of BTAD during the study period. BTAD represented 45.6% of all aortic dissections leading to hospital admission. Incidence for BTAD was 1.62 per 100 000 inhabitants per year. The median survival was 12.7 years [95% confidence interval (CI) 9.63–14.7], 12.4 years (95% CI 10.5–14.4) and 8.6 years (95% CI 7.5–9.7) for patients treated with thoracic endovascular aortic repair (TEVAR), surgery and medical treatment (MT), respectively. Survival was significantly better after TEVAR and surgery, compared to MT only (*P* < 0.001). Age-adjusted survival was significantly better after TEVAR compared to patients treated with MT or surgery (hazard ratio 0.578, 95% CI 0.420–0.794, *P* < 0.001). Aortic-related death was the most common cause of death in all groups (41%).

**CONCLUSIONS:**

The incidence of BTAD seems to be similar in the Finnish population compared to other populational studies. Patients treated with TEVAR had significantly better survival compared to other patients. A high risk for late aortic-related death should be recognized in patients with BTAD.

**Figure:**
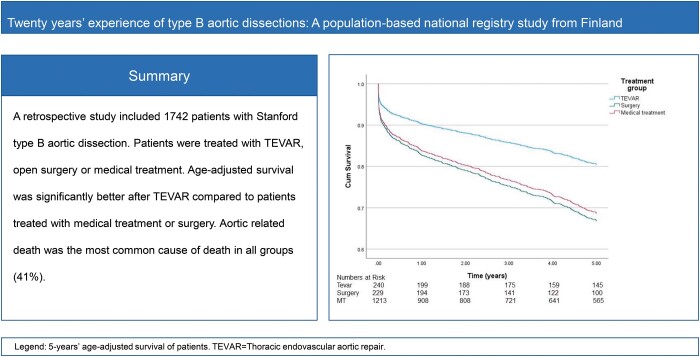

## INTRODUCTION

Aortic dissection (AD) is a life-threatening aortic condition with a reported incidence of 2.5–6 per 100 000 per year [1–5]. Some authors have reported even higher occurrences up to 16 per 100 000 per year [6–8]. Type B aortic dissection (BTAD) is reported to represent ∼22–38% of all ADs [1, 2, 4, 9, 10]. Many academic works concentrate their focus on reporting a combined incidence of AD or type A aortic dissection (ATAD) incidence alone [6, 8] while the incidence of BTAD is rather unknown [1, 2, 3]. Operative treatment of acute ATAD is a golden standard [11], whereas treatment of uncomplicated BTAD remains controversial despite the academic work that has been done recently [12–14]. Recent aortic guidelines propose that complicated BTAD should be treated with endovascular treatment, but the benefits of treating uncomplicated BTAD with thoracic endovascular aortic repair (TEVAR) are rather unclear [15]. There is a consensus that endovascular treatment for patients with uncomplicated BTAD who have high-risk anatomic features may be beneficial and may be considered [15]. Also, understanding of the risk factors for patients receiving a BTAD is limited. Previously reported risk factors include high blood pressure, hereditary connective tissue disorders (HCTD), aortic-arch elongation and enlarged diameter of the aorta [8, 16]. However, there are only a few population-based registry studies concentrating on this matter [2–5]. In addition, knowledge of the long-term survival of patients with BTAD is limited.

Here, we wanted to study the incidence of AD in the Finnish population to gain a greater understanding of the nature of BTAD and its characteristics. The aim was also to study the survival of the patients with BTAD and to identify possible subgroups with impaired prognosis in this patient cohort.

## MATERIALS AND METHODS

### Ethical statement

This study was approved by the institutional review board of Kuopio University Hospital on 11 April 2017 (no. 200/2017). According to the IRB guidelines, the study satisfied the conditions for waiving the requirement for informed consent from individual participants because it involved a retrospective database review.

### Data collection

Data search was made from the nationwide Care Register for Health Care (CRHC), at the Finnish National Institute for Health and Welfare. This register automatically collects discharge data of all hospital admissions in Finland. All patients aged over 15 years with BTAD (ICD-10; I71.01; Stanford B) during the years 2000–2019 were included. All data were collected retrospectively. CRHC collects data including sex, age, length of the hospital stay, diagnose codes (ICD.10), operational codes (Nordic Classification of Surgical Procedures) and the dates of procedures. ICD.10 has been in public use in all hospitals in Finland since 1996. No other diagnose coding system was used during the study period. Data search from the Registry of Death Cause (Statistic Finland) was also made to identify the patients who died before hospital admission. The date and cause of death of patients with BTAD were also collected from the Registry of Death Cause.

CRHC registry has been previously used in similar studies [17]. The reliability of this registry was validated by Ahtela *et al.* [17], who demonstrated that CRHC ICD.10 diagnostic codes were 96.8% specific for the indicated disease infective endocarditis (IE).

The primary outcome of this study was the survival of the patients in the treatment group. The secondary outcome was re-operations. We also wanted to study the age-adjusted survival of patients regarding the treatment group, and a multivariate model was made for this comparison. To find out possible turning points in survival, the Cox-regression model was made for 1–5 and 1–20 years.

### Statistics

All statistical analyses were done with SPSS software (IBM SPSS Statistics for Windows, Version 26.0. Armonk, NY: IBM Corp). Categorical variables are presented as percentages with the number of cases. Continuous variables are presented as means or medians with standard deviation or range. Statistical differences between the groups were pointed out by using cross tabulation with Chi-square or Fisher's exact tests and analysis of variance or the Kruskal–Wallis test. Survival analyses were made by using the Kaplan–Meier method and the Cox-regression model. *P*-value <0.05 was considered statistically significant.

## RESULTS

### Incidence

**Figure 1:**
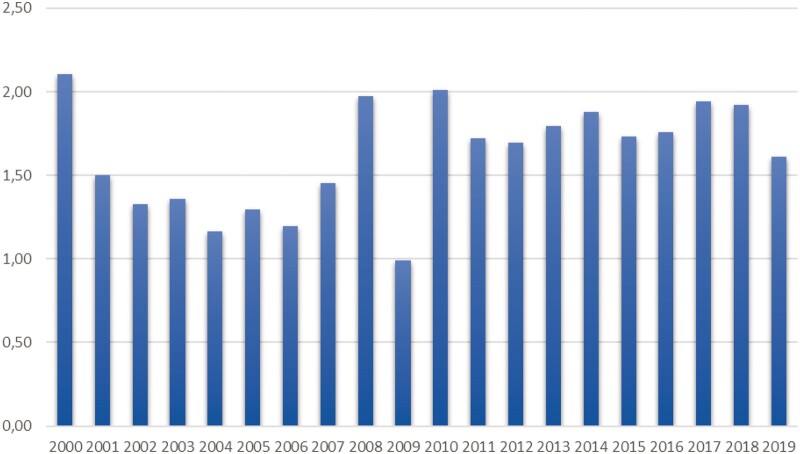
Incidence of type B aortic dissection in Finland 2000–2019 (cases per 100 000/year).

There were 3699 cases of AD leading to hospital admission during the study period (2000–2019), of whom 1682 cases were patients diagnosed with BTAD. When the deaths before the hospital admission were included, there were altogether 1742 patients with BTAD diagnosis between the years 2000–2019. The estimated incidence for BTAD 2000–2019 was 1.62 per 100 000 per year (Fig. 1). BTAD represented 45.6% of all ADs leading to hospital admission. Almost half (831 patients, 47.7%) of the BTAD patients had died during the 2000–2019 period according to the Registry of Death. Only 60 patients (3.4%) had died in the BTAD group before hospital admission, indicating that the majority of the deaths occurred after the diagnosis. A total of 220 (28.5%) patients had AD (ICD.10) as a cause of death.

### Patient characteristics

The mean age of the patients was 66.1 years (15–98), and a large majority (71%) were male gender. Patient characteristics are shown in Table 1. During the years 2000–2019, 229 patients (13.6%) with BTAD were treated with TEVAR, 240 patients (14.3%) were treated with open surgery and 1213 patients (72.1%) were treated with medical treatment (MT). There was no statistically significant difference in sex between the treatment groups (*P* = 0.492). All groups differed in age compared to each other (*P* < 0.001). Patients in the surgery group had a lower mean age (57.6 years) compared to the TEVAR group (64.8 years) and the MT group (68 years) (*P* < 0.001). Patients in the surgery group had a remarkably higher risk for associating ATAD compared to patients in the TEVAR group and the MT group (*P* < 0.001). Also, the need for repair of the ascending aorta was remarkably higher in the surgery group compared to the TEVAR group and the MT group (*P* < 0.001).

**Table 1:** Demographics of the patients treated for type B aortic dissection

	Total	TEVAR group	Surgery group	MT group	*P*-value
*N* (%)	1682 (100)	229 (13.6)	240 (14.3)	1213 (72.1)	
Age, mean (SD, min–max)	66.1 (±13.6, 15–98)	64.8 (±13.5, 15–89)	57.6 (±14.3, 18–84)	68.0 (±12.8, 24–98)	<0.001
Sex, male (%), *n* (%)	1196 (70.8)	170(73.9)	172 (71.4)	854 (70.1)	0.492
Aneurysm of thoracic aorta (%), *n* (%)	36 (2.1)	5 (2.2)	14 (5.8)	17 (1.4)	<0.001
Repair of ascending aorta (%), *n* (%)	277 (16.4)	31 (13.5)	90 (37.3)	156 (12.8)	<0.001
All ATAD (%), *n* (%)	426 (25.2)	52 (22.6)	126 (52.3)	248 (20.4)	<0.001
No ATAD (%), *n* (%)	1261 (74.7)	177 (77.0)	114 (47.3)	970 (79.6)	<0.001
ATAD after BTAD (%), *n* (%)	288 (17.1)	36 (15.7)	83 (34.4)	169 (13.9)	<0.001
Time (median) from BTAD to ATAD (years)	0.58	0.44	1.34	0.36	0.012
ATAD prior to BTAD (%), *n* (%)	138 (8.2)	16 (7.0)	43 (17.8)	79 (6.5)	<0.001

TEVAR group = patients treated with TEVAR, surgery group = patients treated with open surgery and MT group = patients treated with medical treatment.

ATAD: type A aortic dissection; BTAD: type B aortic dissection; MT: medical treatment; TEVAR: thoracic endovascular aortic repair.

### Type B aortic dissection with type A aortic dissection

Of all BTAD patients, 25.3% also had ATAD. In most of these cases (67.6% *n* = 288), ATAD occurred after BTAD and only in 32.4% (*n* = 138) prior to BTAD. The risk for later ATAD was remarkably higher in the surgery group than in the TEVAR or MT groups (*P* < 0.001). The median time from initial BTAD to ATAD was significantly longer in the surgery group compared to the TEVAR and MT groups (*P* = 0.012).

### Aortic surgery and need for re-operations

16.4% of all patients had their ascending aorta replaced. The need for replacement was largest in the surgery group, where 37.3% of patients had replacement of ascending aorta. Re-operations occurred in both TEVAR and surgery groups after the first operation for BTAD. In the TEVAR group, 36 patients (15.7%) had reoperation after primary TEVAR. In the surgery group, 62 patients (25.7%) had reoperation after primary open surgery.

### Survival

**Figure 2:**
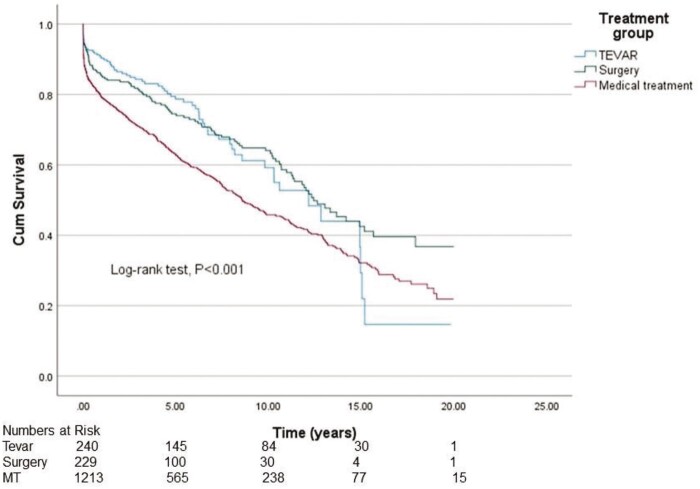
Cumulative Kaplan–Meier estimate of overall survival of patients.

**Figure 3:**
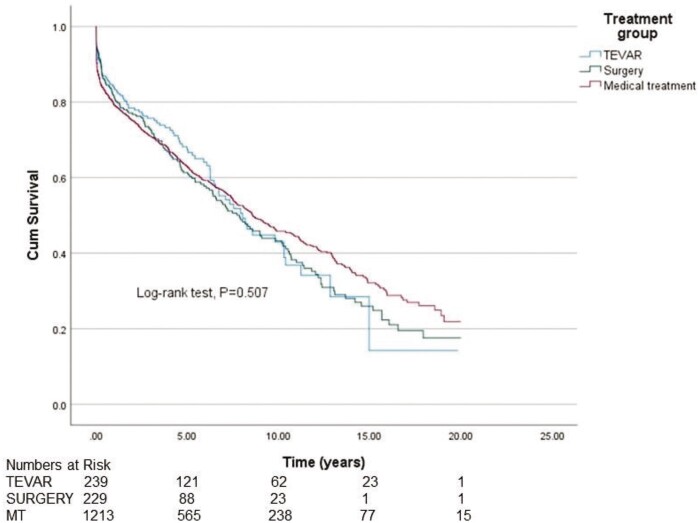
Cumulative Kaplan–Meier estimate of freedom from all-cause mortality and re-operations.

The median survival in the TEVAR, surgery and MT groups was 12.7 years [95% confidence interval (CI) 9.63–14.7], 12.4 years (95% CI 10.5–14.4) and 8.6 years (95% CI 7.5–9.7), respectively, demonstrating significantly better survival in the TEVAR group and the surgery group compared to the MT group (*P* < 0.001) (Fig. 2). There was no statistically significant difference between the groups in ‘freedom from all-cause mortality’ and ‘re-operations’ (*P* = 0.507) (Fig. 3). The 20-year overall survival was significantly better in the TEVAR group and the surgery group compared to the MT group (*P* < 0.001). Because of significant differences in mean age between the groups, we also wanted to study age-adjusted survival. Age-adjusted survival was remarkably better for the TEVAR group compared to the surgery group and the MT group (*P* = 0.023 and *P* = 0.014, respectively).

**Figure 4:**
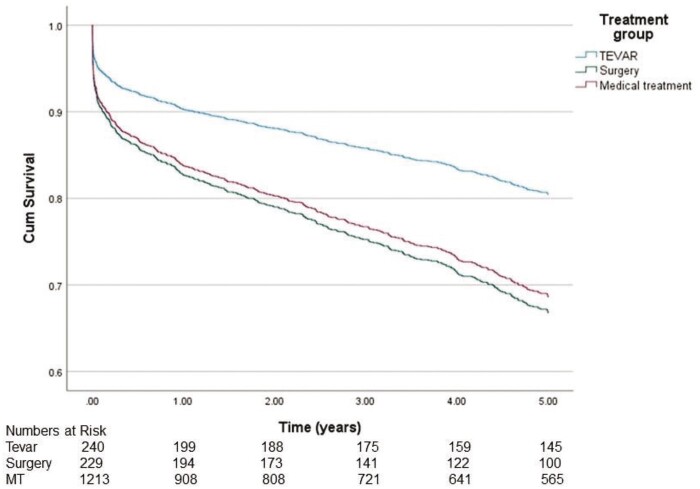
Five-year age-adjusted survival of patients.

**Figure 5:**
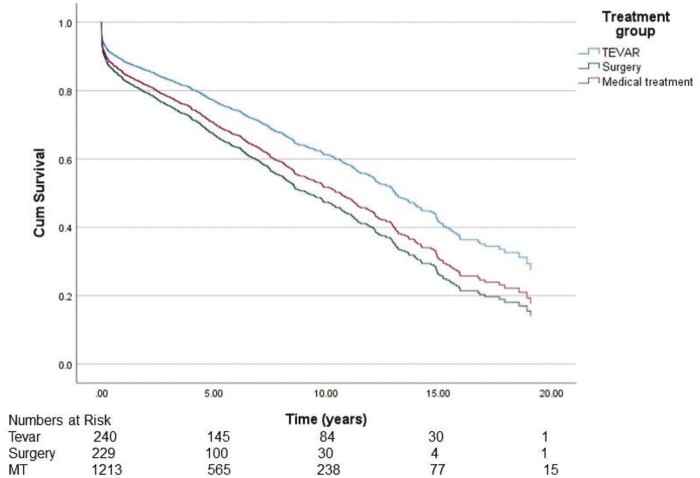
Age-adjusted survival of patients (0–20 years).

To find out possible turning points in survival, we analysed the data in a Cox-regression model for 1–5 years (Fig. 4) and 1–20 years (Fig. 5). Analysis showed that during the first 5 years, survival in the TEVAR group was significantly better compared to the MT group [hazard ratio (HR) 0.578, 95% CI 0.420–0.794, *P* < 0.001]. In contrast, in the surgery group, survival did not significantly differ when compared to the MT group (HR 1.072, 95% CI 0.806–1.425, *P* = 0.630). After 5 years from the diagnosis, the difference in survival did not increase further between the groups (TEVAR group HR 1.360, 95% CI 0.892–2.073, *P* = 0.154 and surgery group HR 1.281, 95% CI 0.919–1.1803, *P* = 0.155, respectively).

### Cause of death

A total of 831 patients died during the follow-up period. This number also includes patients who died before hospital admission. A total of 771 patients died after the hospital admission and diagnosis of BTAD. The causes of death are shown in Table 2. There was a statistically significant difference in the cause of death between the groups (*P* = 0.004). The most common cause of death was aortic-related death (AD or other aortic reasons including rupture of aortic aneurysm and injury of aorta) in all groups (41.1%). Almost one-third of deaths were due to AD. There were less deaths attributed to other cardiovascular deaths in the surgery group compared to both the TEVAR and MT groups. However, aortic-related deaths were 56.9% of all deaths in the surgery group, which was significantly more than in either the TEVAR or MT groups (*P* < 0.001).

**Table 2:** Causes of death in patients with type B aortic dissection

Cause of death	Total	TEVAR group	Surgery group	MT group	*P*-value
All aortic	317 (41.1)	31 (46.3)	58 (56.9)	228 (37.9)	<0.001
Aortic dissection	220 (28.5)	24 (35.8)	37 (36.3)	159 (26.4)	0.047
Other aortic	97 (12.6)	7 (10.4)	21 (20.6)	69 (11.5)	0.030
Cardiovascular (aortic excluded)	202 (26.2)	22 (32.8)	20 (19.6)	160 (26.6)	0.143
Malignancy	101 (13.1)	7 (10.4)	10 (9,8)	84 (14.0)	0.418
Diabetes	2 (0.3)	1 (1.5)	0 (0)	1 (0.2)	0.184
Dementia	15 (1.9)	0 (0)	1 (1.0)	14 (2.3)	0.504
Neurological	30 (3.9)	0 (0)	4 (3.9)	26 (4.3)	0.230
Lung related	41 (5.3)	3 (4.5)	4 (3.9)	34 (5.6)	0.879
Gastrointestinal	21 (2.7)	0 (0)	0 (0)	21 (3.4)	0.056
Sepsis	1 (0.1)	1 (1.5)	0 (0)	0 (0)	0.087
Urinary tract infection	7 (0.9)	0 (0)	0 (0)	7 (1.1)	0.790
Other	34 (4.4)	2 (3.0)	5 (4,9)	27 (4.5)	0.905
Total	771	67 (100.0)	102 (100.0)	602 (100.0)	

TEVAR group = patients treated with TEVAR, surgery group = patients treated with open surgery and MT group = patients treated with medical treatment.

MT: medical treatment; TEVAR: thoracic endovascular aortic repair.

## DISCUSSION

### Incidence

Based on our current findings, the estimated incidence of BTAD was 1.62 per 100 000 per year, which remains at the same level as other studies have reported before [1–5]. However, the share of BTAD (45.6%) of all hospital admissions for AD was higher than previous reports (33.0–38.5%) [1, 2, 4, 9, 10]. The reason for this higher incidence of BTAD may be due to the inclusion of only patients who were admitted to the hospital for AD. Other authors have presented that up to 17.6–48.6% of patients with ATAD die before hospital admission, leading to a situation that a big part of ATAD might not have been included in this comparison [2, 4]. In our study, the estimated incidence of BTAD remained almost at the same level during the 20-year period. This may be because of poorly recognized risk factors for BTAD and a lack of medication or interventions for risk reduction.

### Patient characteristics

Over 70% of patients were male; therefore, it appears that the male gender seems to be an independent risk factor for BTAD. Other authors have also reported male sex to be overrepresented among patients with BTAD [9, 10]. The mean age of the patients was 66.1 years. However, there was a significant difference in age between the groups; patients in the surgery group were younger compared to the TEVAR and MT groups. This difference may be explained by a more active approach of the treatment in young patients with BTAD and that younger patients are more likely to receive open surgery as a primary treatment in order to repair the whole affected aorta in 1 session. Patients having HCTD are also more likely to have AD at a younger age. Replacement of the aorta in these patients may be more challenging and require more often a surgical approach, which may also explain why younger patients received open surgery treatment of BTAD more often. Patients with BTAD are not routinely gene-tested for HCTD in Finland. However, we were able to recognize some patients with HCTD, but because of a lack of routine gene testing, the number of patients with HCTD was too small to be analysed further.

### Aortic events

In this study, we found out that patients with BTAD seem to be at a higher risk of having associated ATAD. The specific reason for the increased risk for ATAD after BTAD remains unknown. However, we believe that this increased risk for ATAD after BTAD is based on aortic wall pathology and other factors, which are recognized risk factors for both ATAD and BTAD [11]. Based on this connection between ATAD and BTAD patients and their mutual risk factors, we believe that patients with ATAD and BTAD are merely representing the same syndrome in different forms rather than 2 different syndromes. Furthermore, in our study, the risk for ATAD after BTAD seems to be much higher than another study has shown [18]. Therefore, we suggest that patients with BTAD should be followed with a standardized follow-up protocol with annual imaging of the aorta to identify patients with progressive aortic disease and who are in need of preventive replacement of the ascending aorta.

### Survival

Age-adjusted survival was significantly better in the TEVAR group than in the surgery group. Many other studies have also illustrated better outcomes for TEVAR-treated patients compared to patients treated with surgery [19, 20]. Because of the retrospective nature of this study, we do not know the exact characteristics of each patient’s AD. Therefore, we hypothesize that this difference in survival between the groups can also be due to the more complex nature of the aortic disease in the surgery group. Patients requiring arch and descending aortic replacement are more challenging and are known to have higher mortality and morbidity when compared to endovascular or medically treated patients and, therefore are not directly comparable. This more complex aortic morphology may be an independent factor for impaired survival among the patients in this patient group. There was no difference in ‘freedom of all-cause mortality’ and ‘re-operations’ between the groups. This indicates that reoperation improved patient survival in the TEVAR group and in the surgery group compared to the MT group.

There was also a significant difference in age-adjusted survival between TEVAR and MT groups. This study suggests that endovascular TEVAR treatment may provide an independent survival benefit for patients receiving this treatment over other MTs. Other studies have also had similar outcomes for the patients treated with TEVAR compared to MT [14, 21, 22]. However, some authors have presented results with no difference in overall survival in 0–5 years between the TEVAR and MT groups [23]. That might be due to patient selection or a limited study population. Based on our findings, the benefits of TEVAR treatment in patients with BTAD should be considered and further studied.

### Causes of death

Aortic-related cause of death was found to be the most common cause of death in all groups. However, aortic-related deaths formed 56.9% of all deaths in the surgery group, which was significantly more than in both the TEVAR and MT groups (*P* < 0.001). This difference between the groups may be another indicator of the more complex morphology of aortic disease in patients receiving surgical treatment. The lower aortic-related death ratio in the MT group could be explained by patients with higher mean age and a larger proportion of age-related causes of death. Because of the relatively high number of aortic-related causes of death in all groups, the need for a more intensive and standardized patient follow-up protocol should be considered to identify patients who are at the highest risk for having new aortic events after BTAD. There is a need for high-quality randomized controlled trials for young patients having BTAD to find out possible differences in long-term survival between TEVAR treatment and open surgery in this patient cohort.

### Limitations

The study had several limitations. First of all, it is a retrospective register study. Although CRHC automatically collects all discharge data of all hospital admissions, there may be some inaccuracies in the diagnosis codes and other data. Also, challenges in diagnostics, particularly in differentiating the modes of ADs, may cause some inaccuracy in the final diagnosis. Another limitation of the registry is that the amount of gathered data is limited, and the specific information considering the dissection (acute, subacute, chronic) is not gathered. We recognize that more accurate data collection by register authorities would have been valuable and could have helped us to make more specific comparisons between the subgroups and provide accurate data on the acuity and chronicity of each AD. Although the register gathers some data regarding prescribed medication, inaccuracies in medication made sub-analysis impossible to study the usage of medication for high blood pressure in these patients. We are aware that patient groups may differ each other’s in comorbidities and therefore they are not directly comparable to each other. Unfortunately collecting other diagnoses than main diagnose to CRHC registry is not mandatory by Finnish law and therefore reporting other diagnoses than main diagnose of each hospital admission is completely relying how accurately other diagnoses have been recorded to the patient’s medical records. We made sub-analysis of patient’s comorbidities regarding the treatment group, but we found that a remarkable share of the patients lacked necessary information on other diagnoses than main diagnose (AD) and therefore results were too insufficient to be presented in this study. Because of retrospective nature of this register study, we do not have all the information regarding how the decisions between different treatment modalities were done. We believe that during 2000–2019 patients were treated according to the current guidelines and patients who had uncomplicated BTAD were most likely treated with MT. Also, patients who were either treated with TEVAR or open surgery were very like to have complicated BTAD and therefore were treated with invasive treatment. We are also aware that there may be some differences in practice regarding the choice of treatment between the centres. Part of the patients with BTAD who were treated with MT was treated in hospitals which are not TEVAR centres. Therefore, the tendency of these patients receiving MT as a primary treatment may be higher compared to patients whom we treated in TEVAR centres.

## CONCLUSION

The estimated incidence of BTAD in the Finnish population is similar to that found in other registries. Male gender seemed to be an independent risk factor for BTAD. There is a strong relationship between BTAD and ATAD. Endovascular TEVAR treatment seemed beneficial in terms of survival and should be further studied in prospective randomized trials. In addition, prospective randomized trials are needed to highlight the superiority of TEVAR treatment over MT, especially in patients with uncomplicated BTAD. Because of the high risk for later aortic events, patients with BTAD should be monitored closely with regular follow-up protocol. A high risk for later aortic-related cause of death should be recognized in all AD patients.

## Data Availability

The data underlying this article were provided by the Finnish National Institute for Health and Welfare under license/by permission. Data will be shared on request to the corresponding author with the permission of the Finnish National Institute for Health and Welfare.

## ABBREVIATIONS

AD: Aortic dissection
ATAD: Type A aortic dissection
BTAD: Type B aortic dissection
CI: Confidence interval
CRHC: Care Register for Health Care
HCTD: hereditary connective tissue disorder
MT: Medical treatment
TEVAR: Thoracic endovascular aortic repair
